# A dataset consisting of a two-year long temperature and sound speed time series from acoustic tomography in Fram Strait

**DOI:** 10.1016/j.dib.2022.108118

**Published:** 2022-04-01

**Authors:** F. Geyer, H. Sagen, B. Dushaw, A. Yamakawa, M. Dzieciuch, T. Hamre

**Affiliations:** a Nansen Environmental and Remote Sensing Center, Jahnebakken 3, Bergen 5007, Norway; b Scripps Institution of Oceanography, UC San Diego, 9500 Gilman Drive, La Jolla, CA 92093, United States of America

**Keywords:** Underwater acoustics, Oceanography, Arctic ocean, NetCDF

## Abstract

Acoustic tomography systems provide an integrated, synoptic measurement of ocean temperature. By recording the time it takes for a sound signal to travel from a sound source to a receiver, the depth- and range-average sound speed along the geodesic path between the sound source and the receiver can be obtained through inversion. Sound speed and ocean temperature are empirically related; salinity plays a negligible role.

The ACOBAR acoustic tomography experiment in central Fram Strait was carried out from September 2010 to September 2012. It consisted of 3 moorings with sound sources and receivers forming a triangle, and one mooring with only receivers in the middle. The steel-sphere flotation of the northernmost mooring imploded in the start of the experiment, so that mooring was not recovered. The three remaining moorings formed a smaller triangle that provided travel time measurements along three paths. Measurements were taken 8 times a day for two of the paths, 8 times every other day for the other paths. The distances covered by the acoustic measurements are 188 – 201 km.

Complex data processing was used to determine peaks in the acoustic arrival coda and to correct them for mooring motion and clock drift; travel-time accuracy is O(10) ms. The travel time measurements were inverted to obtain range-depth average sound speed using a statistical approach. The sound speed obtained from each section was then converted to mean ocean temperature.

The mean ocean temperature data are published as a set of 8 NetCDF files, compliant with Climate and Forecast (CF) [1] and OceanSITES metadata conventions [2]. Each file contains one year of measurements from one of the sections. The files contain the ocean temperature data, together with theoretical and statistical error estimates and metadata such as discovery metadata and adequate-use metadata. Each data point is provided with a statistical quality measure and a quality flag based on this measure.

## Specifications Table

**Table:** 

Subject	Oceanography
Specific subject area	Range- and depth-averaged ocean temperature derived from ocean sound speed obtained using acoustic tomography.
Type of data	Table, Figures, NetCDF data files
How data were acquired	Acoustic travel times were measured between sources and receivers placed on fixed moorings in the Fram Strait. Acoustic travel times were inverted to obtain sound speed [3], which was converted to ocean temperature. Acoustic tomography, inversion
Data format	Analyzed
Description of data collection	The data were obtained by inverse methods applied to measured under-water acoustic travel times between acoustic source and receiver moorings
Data source location	Data correspond to acoustic sections between 3 moorings in Fram Strait between Svalbard and Greenland. Mooring A at 77°53.9912′N 008°44.8132′E Mooring B at 78°09.6527′N 004°14.5812′W Mooring D at 78°53.7063′N 002°19.6998′E
Data accessibility	Repository name: Norwegian Marine Data centre Data identification number: https://doi.org/10.21335/NMDC-NERSC-1,237,754,860 Direct URL to data: https://thredds.nersc.no/thredds/catalog/arcticData/framStrait/ACOBAR_soundscapeL2/catalog.html
Related research article	[5] Geyer, F., Sagen, H., Cornuelle, B., Mazloff, M. R., Vazquez, H. J. (2020), Using a regional ocean model to understand the structure and variability of acoustic arrivals in Fram Strait, J. Acous. Soc. Am., 147 (2), 1042–1053, doi:10.1121/10.0000513. [6] Dushaw, B. D., and Sagen, H. (2016), A comparative study of the properties of moored/point and acoustic tomography/integral observations of Fram Strait using objective mapping techniques, J. Ocean. Atmos. Tech., 33, 2079–2093, doi:10.1175/JTECH-D-15-0251.1. [10] Sagen, H., Dushaw, B. D., Skarsoulis, E. K., Dumont, D., Dzieciuch, M. A., Beszczynska-Möller, A. (2016), Time series of temperature in Fram Strait determined from the 2008–9 DAMOCLES acoustic tomography measurements and an ocean model, J. Geophys. Res., 121, doi:10.1002/2015JC011591. [13] Dushaw, B. D., Sagen, H., and Beszczynska-Möller, A. (2016), On the effects of small-scale variability on acoustic propagation in Fram Strait: The tomography forward problem, J. Acoust. Soc. Am., 140, 1286{1299, doi::10.1121/1.4961207.

## Value of the Data

• Range- and depth-integrated temperature and sound speed data in Fram Strait between Svalbard and Greenland are useful because they provide an integrated measure of these variables in this highly dynamic and important ocean area, where point measurements are only representative of small ocean areas.
• This dataset is beneficial for oceanographers trying to understand the complex dynamics and oceanographic variability in Fram Strait. It is of special use to researchers working with data assimilation to provide realistic state estimates of the North Atlantic and Arctic Ocean and the exchanges between them.
• The data give important insights for future Arctic observing systems, for the design of acoustic observing systems and the inclusion of integrated measures into data assimilation systems.
• The additional value is in the complementary information contained in integrated ocean measurements relative to point measurements. Dushaw and Sagen [6] have demonstrated in an idealized model study how combining point measurements with integrated measurements increases the total knowledge of the ocean.
• The datasets include quality flags for each raw data point and smoothed data in addition to the raw data. The full raw data provide understanding of the acoustic and oceanographical complexity in the Fram Strait, while good data filtered by the flags and the smoothed data are useful for models.

## 1 Data Description

**Fig. 1:**
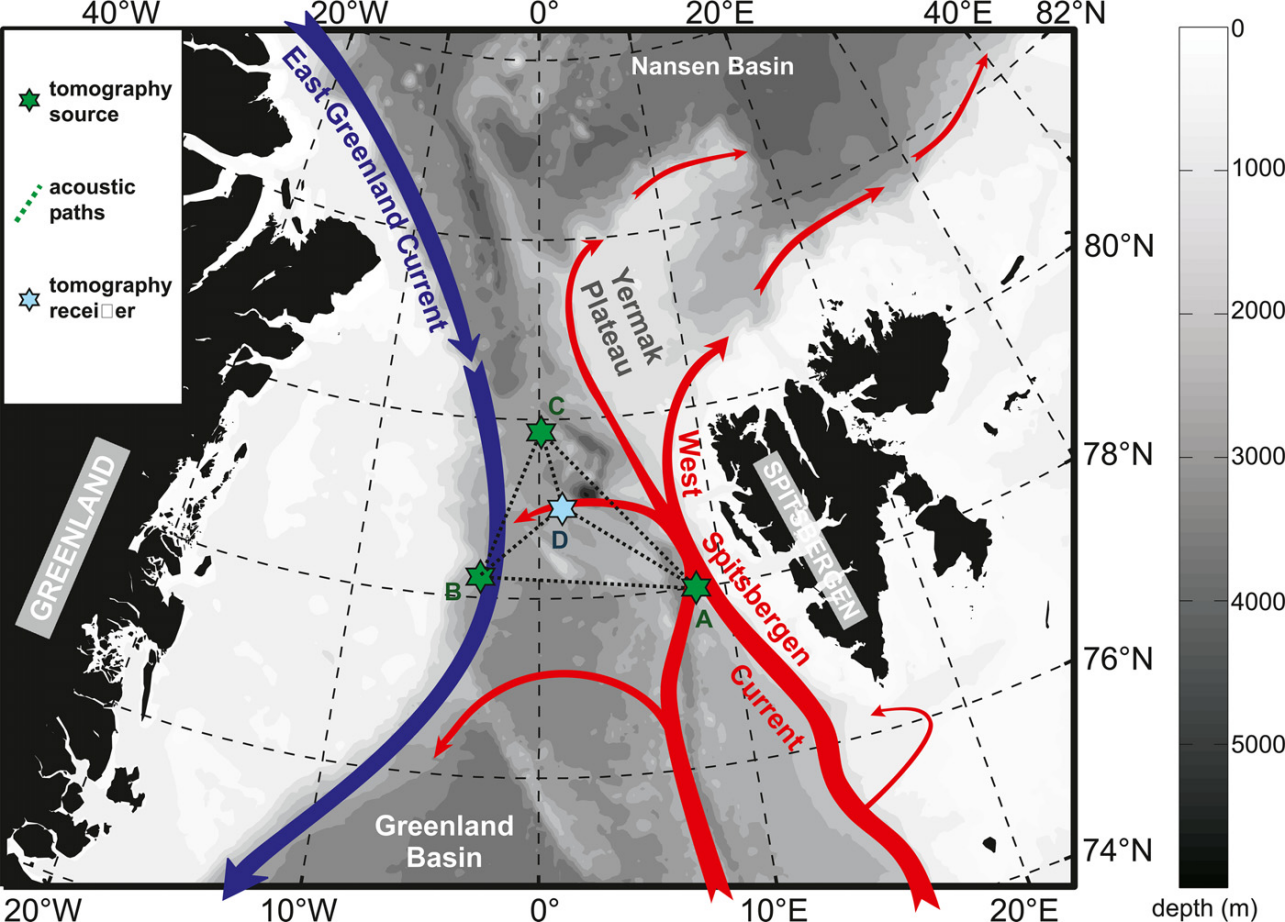
Location of the ACOBAR acoustic tomography experiment in the Fram Strait between Greenland and Spitsbergen (figure adapted from [4] under CC BY license).

This dataset consists of several time series of depth- and range-averaged temperature and sound speed for 3 separate acoustic sections in the central Fram Strait (Fig. 1). One of these sections was measured independently in both directions, so in effect there are 4 sections in total [4,5]. Table 1 lists the 3 acoustic moorings in the experiment and the distances between them. The times series cover the period from September 2010 to May/July 2012 with a break in the time series during the summer 2011, when the moorings were recovered, equipped with new batteries and redeployed. The end date of the time series in 2012 was determined by the available energy in batteries on the respective moored instruments.

**Table 1:** Ranges (km) between the mooring locations.

	A	B	D
A	0	301.11	181.62
B		0	167.09
D			0

**Fig. 2:**
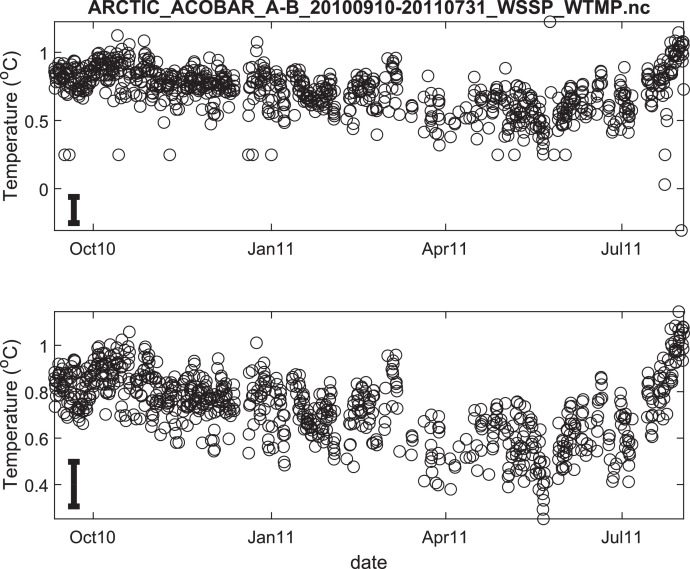
Temperature time series ACOBAR section A-B 20,100,910–20,110,731. The date is given in the format month-year (mmmyy). Upper panel: all data, lower panel: data flagged as “probably good data”). The statistical error estimate is indicated by the error bar in the lower left corner.

**Fig. 3:**
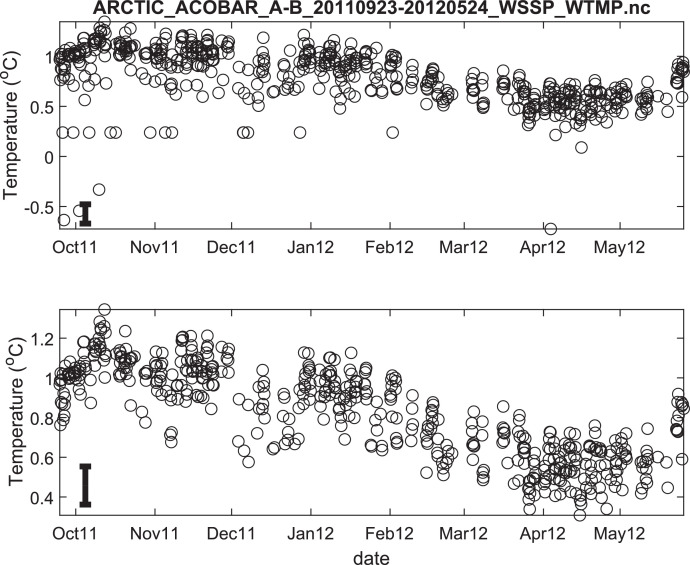
Temperature time series ACOBAR section A-B 20,110,923–20,120,524. The date is given in the format month-year (mmmyy). Upper panel: all data, lower panel: data flagged as “probably good data”). The statistical error estimate is indicated by the error bar in the lower left corner.

**Fig. 4:**
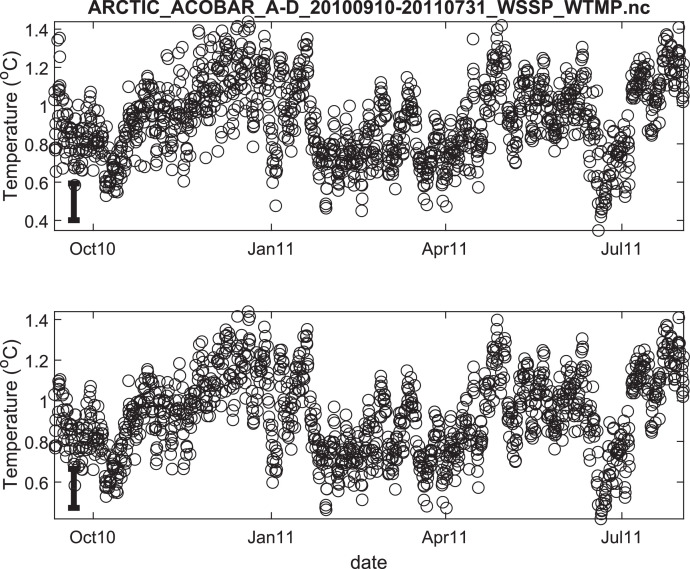
Temperature time series ACOBAR section A-D 20,100,910–20,110,731. The date is given in the format month-year (mmmyy). Upper panel: all data, lower panel: data flagged as “probably good data”). The statistical error estimate is indicated by the error bar in the lower left corner.

**Fig. 5:**
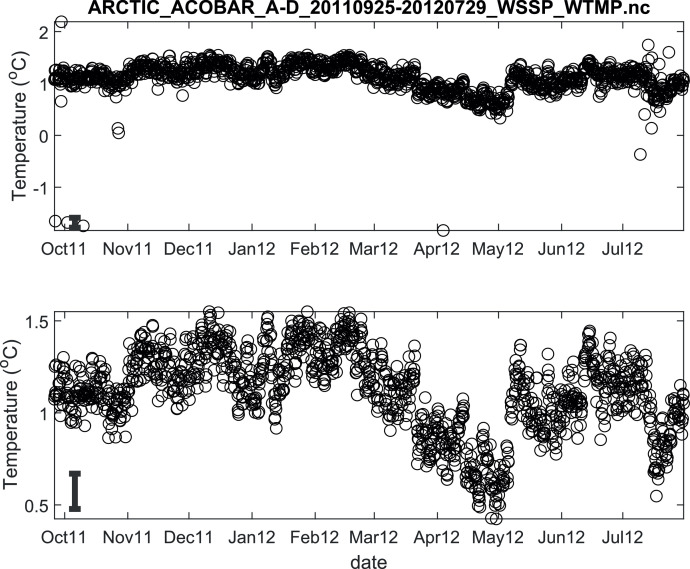
Temperature time series ACOBAR section A-D 20,110,925–20,120,729. The date is given in the format month-year (mmmyy). Upper panel: all data, lower panel: data flagged as “probably good data”). The statistical error estimate is indicated by the error bar in the lower left corner.

**Fig. 6:**
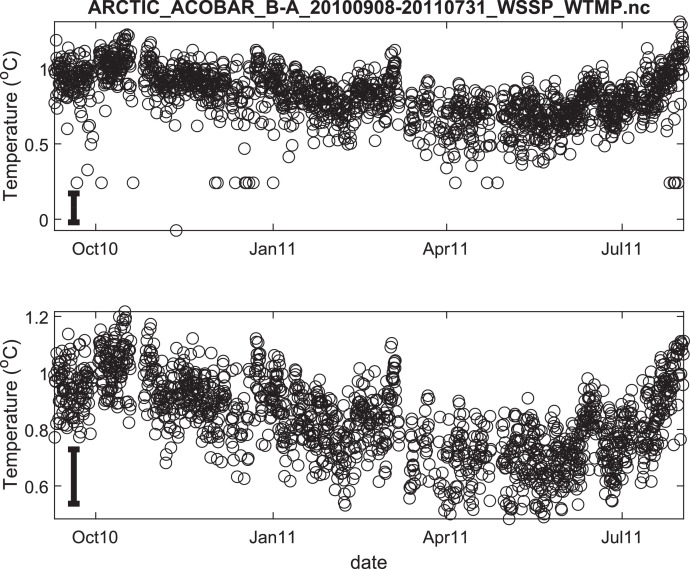
Temperature time series ACOBAR section B-A 20,100,908–20,110,731. The date is given in the format month-year (mmmyy). Upper panel: all data, lower panel: data flagged as “probably good data”). The statistical error estimate is indicated by the error bar in the lower left corner.

**Fig. 7:**
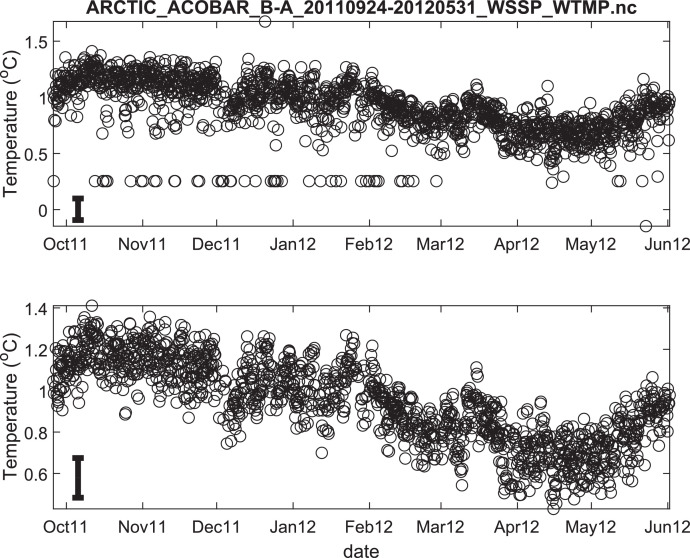
Temperature time series ACOBAR section B-A 20,110,924–20,120,531. The date is given in the format month-year (mmmyy). Upper panel: all data, lower panel: data flagged as “probably good data”). The statistical error estimate is indicated by the error bar in the lower left corner.

**Fig. 8:**
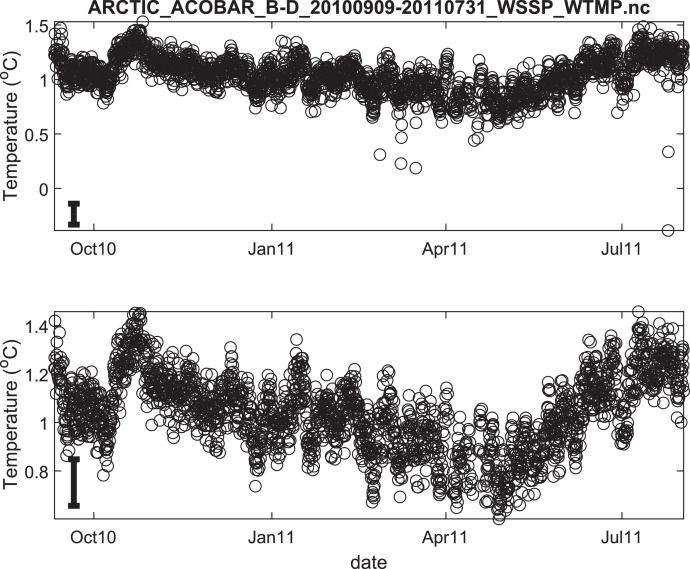
Temperature time series ACOBAR section B-D 20,100,909–20,110,731. The date is given in the format month-year (mmmyy). Upper panel: all data, lower panel: data flagged as “probably good data”). The statistical error estimate is indicated by the error bar in the lower left corner.

**Fig. 9:**
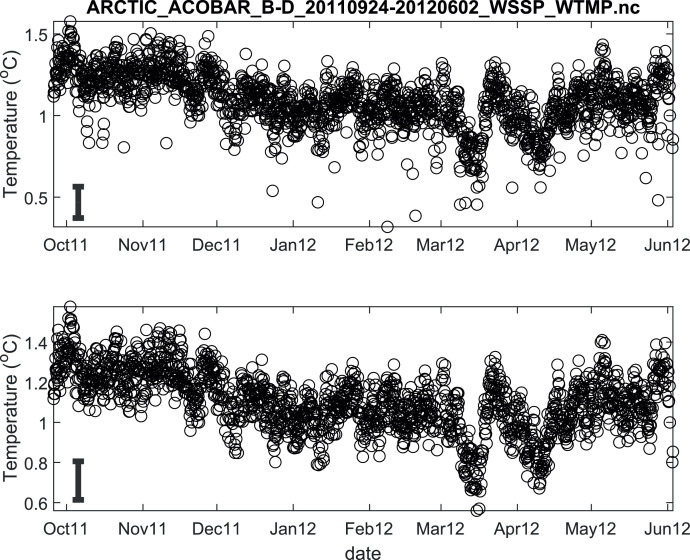
Temperature time series ACOBAR section B-D 20,100,924–20,120,602. The date is given in the format month-year (mmmyy). Upper panel: all data, lower panel: data flagged as “probably good data”). The statistical error estimate is indicated by the error bar in the lower left corner.

The published data files are listed in Table 2. Because the acoustic moorings were recovered and redeployed in September 2011, data for each year are provided in separate files. Thus, there are 8 data files in total. Each file contains estimates of depth- and range-averaged ocean sound speed and of depth- and range-averaged temperature (Fig. 2, Fig. 3, Fig. 4, Fig. 5, Fig. 6, Fig. 7, Fig. 8, Fig. 9). The estimates are derived from the acoustic travel times along each section by using inverse methods (see method chapter). The range average value applies to the length of each acoustic section as listed in Table 1. The depth average is over the upper 1000 m of ocean.

**Table 2:** Published data files in data repository.

Data File	Content
ARCTIC_ACOBAR_B-D_20,110,924–20,120,602_WSSP_WTMP.nc	Ocean sound speed and temperature estimates for acoustic section B-D, second deployment (2011/09/24–2012/06/02)
ARCTIC_ACOBAR_B-D_20,100,909–20,110,731_WSSP_WTMP.nc	Ocean sound speed and temperature estimates for acoustic section B-D, first deployment (2010/09/09–2011/07/31)
ARCTIC_ACOBAR_B-A_20,110,924–20,120,531_WSSP_WTMP.nc	Ocean sound speed and temperature estimates for acoustic section B-A, second deployment (2011/09/24–2012/05/31)
ARCTIC_ACOBAR_B-A_20,100,908–20,110,731_WSSP_WTMP.nc	Ocean sound speed and temperature estimates for acoustic section B-A, first deployment (2010/09/08–2011/07/31)
ARCTIC_ACOBAR_A-D_20,110,925–20,120,729_WSSP_WTMP.nc	Ocean sound speed and temperature estimates for acoustic section A-D, second deployment (2011/09/25–2012/07/29)
ARCTIC_ACOBAR_A-D_20,100,910–20,110,731_WSSP_WTMP.nc	Ocean sound speed and temperature estimates for acoustic section A-D, first deployment (2010/09/10–2011/07/31)
ARCTIC_ACOBAR_A-B_20,110,923–20,120,524_WSSP_WTMP.nc	Ocean sound speed and temperature estimates for acoustic section A-B, second deployment (2011/09/23–2012/05/24)
ARCTIC_ACOBAR_A-B_20,100,910–20,110,731_WSSP_WTMP.nc	Ocean sound speed and temperature estimates for acoustic section A-B, first deployment (2010/09/10–2011/07/31)

The acoustic source on mooring A transmitted its signal once every 3 h. The acoustic source on mooring B transmitted every 3 h on each second day. Therefore, the data files for the acoustic sections A to B and A to D contain 8 sound speed and temperature estimates a day, while the sections B to A and B to D provide 8 estimates every second day. The dataset also contains a smoothed estimate for the temperature time series.

In addition to the temperature and sound speed time series, the data files provide a quality variable, which is the squared-weighted misfit between the data and the smoothed time series. A quality flag based on this quality variable classifies each data point as either “probably good data” and “bad data” according to OceanSITES recommendations [2].

## 2 Experimental Design, Materials and Methods

### 2.1 Overview

The ACOBAR acoustic tomography measured acoustic travel times for 3 different sections in central Fram Strait from September 2010 to May/July 2012. For the section between moorings A and B reciprocal travel times were measured, resulting in separate data sets called section A to B and section B to A. The data processing required to obtain the travel times is described in Sagen et al. [4]. Below we describe the methods used to calculate the temperature and sound speed data presented in this paper from those acoustic travel times. In general, this method consists of an inverse model which is used with a reference ocean as a starting point to estimate the ocean sound speed field consistent with the measured acoustic travel times. This ocean sound speed field is averaged over the range of the respective acoustic section and a depth of 0–1000 m, which is the main depth range sampled by the measured acoustic rays. Finally, the range- and depth-averaged sound speed is converted to range- and depth-averaged ocean temperature.

This method chapter is a review of all the steps necessary to calculate ocean sound speed and temperature from the measured acoustic travel times using the inverse method. It aims to give sufficient guidance to enable the replication of the processing chain that was employed for the calculation. While all the parts of the processing have been published before, the explanations have occurred in a piecemeal fashion in separate publications that focused on the separate steps of the processing. Here a succinct review of these elements is provided, brought together from these earlier publications. They are augmented with what hopefully will be helpful instructions for any researcher that intends to replicate the processing of this dataset.

The processing chain consists of the following parts:

• Calculate a reference ocean, that serves as an initial guess for the inverse. In Fram Strait this reference ocean needs to include a semi-empirical model for the small mesoscale variability, otherwise the inverse could not predict the measured travel times adequately. This step is presented in sub-chapter 2 below.
• Calculate acoustic travel times for the acoustic sections using the reference ocean. Appropriately choose calculated rays and associated travel times from the reference ocean; choose acoustic arrivals, which should not include bottom-reflected acoustic rays, and associated measured travel times. The comparison of those serves as the basis for the inverse model. This step is presented in sub-chapter 3 below.
• Set up the inverse model: a numerical model of the ocean, which consists of sets of horizontal and vertical functions with wavenumber spectra that determine their relative contributions. This ocean model can be combined with objective mapping techniques (inverse) to solve for the sound speed sections that correctly predict the measured travel times. This step is presented in sub chapter 4 below.
• Calculate the inverse and verify that solution by calculating the new predicted acoustic travel times. This step is presented in sub-chapter 5 below. For the convenience of the reader, we try to present this in a step-by-step “cookbook style” detailing all the settings chosen to calculate the data set presented here.
• Calculate the range-and-depth averaged estimates of ocean sound speed and ocean temperature. This step is presented in sub-chapter 6 below.

### 2.2 Reference ocean

The reference ocean is calculated as the sum of an ocean climatology and an empirical model for small-scale ocean variability in Fram Strait. The 2009 World Ocean Atlas (WOA09) by the National Oceanic And Atmospheric Administration (NOAA) was used as the ocean climatology [3]. An empirical model was set up to describe the small-scale thermal variations in Fram Strait. It statistically describes the small-mesoscale variability in Fram Strait, which dominates the small-scale variations and explains the stochastic scattering observed in the acoustic measurements [13]. These are presented in the following subchapters.

#### 2.2.1 Reference ocean: climatological ocean

Temperature and salinity from the 2009 world ocean atlas (WOA09) [7,8] were used to calculate a sound speed climatology along the acoustic sections of the ACOBAR acoustic tomography experiment. The Del Grosso sound speed equation was used for this calculation. The ocean bathymetry along the ACOBAR acoustic paths was derived from the International Bathymetric Chart of the Arctic Ocean data base (IBCAO), with a 2 min horizontal resolution [9].

#### 2.2.2 Reference ocean: the small-scale contribution

The empirical ocean model used to construct statistical representations of the small-mesoscale ocean variability in Fram Strait is constructed in the same way as the ocean model employed in the inverse calculation of ocean sound speed from the acoustic travel times [10,11], with updated settings (see sub chapter 4). The ocean variability is assumed to be separable into vertical and horizontal functions. Therefore, the model is composed of vertical modes and horizontal sinusoids.

$$F_{S}\left(r,z\right)=\sum_{i=1}^{N_{V}}V_{i}\left(z\right)\left[A_{i0}^{0}+\sum_{j=1}^{N_{H}}\left(A_{ij}^{1}cos\left(K_{j}r\right)+A_{ij}^{2}sin\left(K_{j}r\right)\right)\right]=\sum_{n=1}^{N}A_{n}P_{n}\left(r,z\right).$$

The vertical modes, *V_i_(z)*, are empirical orthogonal functions (EOFs) of a vertical covariance matrix, which is tuned to fit the vertical covariances observed in Fram Strait on the appropriate scales of variability. For small-mesoscale variability, a covariance matrix in the vertical was set up in the following form:

$$C\left(z_{1},z_{2}\right)=4e^{\frac{-\left(z_{1}+z_{2}\right)}{550}}e^{\frac{{\left(z_{1}-z_{2}\right)}^{2}}{10\left(z_{1}+z_{2}\right)}}.$$

The vertical e-folding scale of variance is 275 m. It can be calculated by setting *z_1_* *=* *z_2_*. Fifty vertical EOFs computed from a Singular Value Decomposition (SVD) of the covariance were used for the empirical model of small-mesoscale ocean variability.

The wavenumber spectrum of horizontal variability used for the empirical description of small-mesoscale ocean variability was in the form:

$$S_{ij}=\frac{w_{i}}{K_{j}^{2}+K_{0i}^{2}}=\langle A_{n}^{2}\rangle =W_{n}.$$

This form assumes an exponential falloff of the spatial covariance with horizontal distance. 1500 horizontal wavenumbers were used. These were defined relative to the length of each acoustic section as *K_j_* *=* *2πj/(1.5* *L),* for the jth vertical mode. K_0i_ was defined ad hoc as K_0i_ *=* K_10j+20_ to give a simulated variability that roughly approximates the expected length scales. Such length scales correspond to the rossby radius of deformation in the region O(4–8 km) for the lowest-order vertical EOFs. Higher-order vertical EOFs have shorter horizontal scales than lower-order EOFs. Using 1500 wavenumbers ensures that sufficiently small horizontal scales are simulated by the empirical model. The overall levels *w_i_*, are set by the EOF spectrum. The resulting variances of the amplitudes of the 2D functions obtained by combining the vertical EOFs and the horizontal wavenumber spectra can then be used to produce statistically consistent snapshots of the small mesoscale ocean.

For these mesoscale snapshots, the standard deviations of the amplitudes of the 2D function were multiplied by a random number with Gaussian distribution and standard deviation 1 to obtain random amplitudes for the 2-D functions:

$$A_{n}=\sqrt{W_{n}}p_{n}.$$

These random, but correctly scaled amplitudes were then applied to each 2-D function in the equation for *F_S_(r, z)*. This results in a simulated snapshot of random small-mesoscale ocean variability. The resulting small-scale sound speed variations are roughly consistent with the high-frequency variations of sound speed observed by the AWI (Alfred Wegener Institute) Moored Array situated at 78°50′N in Fram Strait.

### 2.3 Quantification of travel time differences

It was assumed that the acoustic arrivals, which form the main arrival pulse in Fram Strait acoustic tomography measurements, derive from acoustic rays that have been randomly scattered by small-scale variations of ocean sound speed. Therefore, the general approach for the quantification of the differences between measured and modeled acoustic travel times was to not try to identify specific arrivals, but instead to randomly assign rays to measured acoustic arrival peaks [10]. It is therefore important to correctly identify the main arrival pulse, and to exclude later bottom reflected arrivals and the occasional random false pulse detection. The arrival peak with the largest amplitude is assumed to occur within the main pulse. Travel times arriving earlier or later than 150 milliseconds (ms) from this largest-amplitude travel time peak were excluded from the processing. These criteria were sufficient in nearly all cases to exclude bottom reflected arrivals [5], and only peaks within the main arrival pulse remained for use in the inversion. After this filtering the remaining peaks were sorted by amplitude. Typically, there were 5–10 remaining peaks available for the inversion, and ten peaks was the maximum number of peaks used.

These measured arrival peaks then had to be compared to modeled or predicted arrivals. These were produced by calculating the eigenrays between sound source and receiver using the reference ocean as a range-dependent sound speed field along the acoustic path. The computed eigenrays were sorted by the absolute value of the ray angle from the horizontal at the sound source. If N measured arrival peaks were available from the filtering described above, then these were assigned to the first N computed eigenrays. Hereby the measured arrival with the largest amplitude was assigned to the eigenray with the smallest source angle, the measured arrival with the second-largest amplitude was assigned the eigenray with the second-smallest source angle, etc.

To account for the randomness in the assigning of measured arrival peaks and modeled eigenrays, the travel time data were assumed to have an uncertainty of 100 ms. This uncertainty is much larger than the usual uncertainty of individual measured ray arrivals (approx. 10 ms). It rather represents the average width of the main arrival pulse. Despite this large uncertainty the inverse estimates will still be sufficiently accurate because of the large number of randomly assigned data points used to calculate each inverse estimate [10].

### 2.4 The inverse model

The inverse method uses a weighted least-squares fit of a model to a data set, using the associated model and data parameter statistics. In this case the inverse fits a numerical ocean model to the measured acoustic travel time data to obtain sound speed estimates for the ocean section measured by and the acoustic tomography experiment [12,6]. The ocean model used for the inversion is set up in a very similar manner to that of the model constructed in sub-chapter 2 to simulate small-scale ocean variability. The main differences are in the horizontal and vertical scales, since the inverse aims to obtain a smoothed estimate for sound speed. In short, the empirical model in sub-chapter 2 was constructed to describe small-mesoscale variability in the ocean, while aim of the inverse model is to correct the large-scale variability of sound speed to match the observed acoustic travel times.

Like in sub-chapter 2, we assumed the horizontal and vertical variability were separable. The ocean state of a scalar variable such as sound speed *F(r, z)* can be modeled as a linear superposition of two-dimensional (2-D) functions of horizontal distance *r* and depth *z*. The ocean model used for the inversion consists of a truncated Fourier series, that is sines and cosines, in the horizontal, and a small set of empirical orthogonal functions (EOFs) in the vertical:

$$F_{SI}\left(r,z\right)=\sum_{i=1}^{N_{V}}V_{i}\left(z\right)\left[A_{i0}^{0}+\sum_{j=1}^{N_{H}}\left(A_{ij}^{1}cos\left(K_{j}r\right)+A_{ij}^{2}sin\left(K_{j}r\right)\right)\right]=\sum_{n=1}^{N}A_{n}P_{n}\left(r,z\right).$$

#### 2.4.1 Horizontal functions

The horizontal function is constructed as a linear superposition of sinusoids, that is, horizontal variability was modeled using a truncated Fourier series. The horizontal wavenumbers *K_j_* *=* *2πj /(1.5* L*),* for vertical mode j, are defined relative to the range of the Fram Strait acoustic sections. The factor of 1.5 is used to avoid periodicity effects at the ends of the section. If there are *N_V_* vertical empirical orthogonal functions and *N_H_* horizontal wavenumbers, then a total of *N* *=* *N_V_ (2N_H_* *+* *1)* 2-D functions comprise the ocean model for the inversion. With the model employed here, *N_V_* was 5, while *N_H_* was 10, giving a total of 105 2-D functions.

#### 2.4.2 Vertical functions

The vertical functions employed for the inverse model were empirical orthogonal functions (EOFs) of a synthetic covariance function. The synthetic covariance function used for the inverse model was:

$$C\left(z_{1},z_{2}\right)=20e^{\frac{-\left(z_{1}+z_{2}\right)}{750}}e^{\frac{-{\left(z_{1}-z_{2}\right)}^{2}}{100\left(z_{1}+z_{2}\right)}}$$

The variance of sound speed at the surface was assumed to be about 20 (m s^−1^)^2^, and the e-folding scale of variance with depth to be 350 m. This covariance was subsequently adjusted near the surface. The variances within 40 m of the surface were increased by an adjustment factor to model the ubiquitous, near-surface mixed layer found in Fram Strait [6]. Its specific form was:

$$\sqrt{1+2\cdot \left(1-tanh\left(0:.2:5\right)\right)},$$

Corresponding to depths from 0 to 250 m at 10 m intervals. With this form, the surface value of the covariance was increased by a factor 1.73^2^. The vertical parameterization described here approximates the observed variance as a function of depth and vertical correlation length to as observed by the AWI Moored Array in Fram Strait. The singular value decomposition of this covariance gives the EOFs that model the vertical variability.

#### 2.4.3 Wavenumber spectrum

The form for the wavenumber spectrum, which sets the assumed horizontal length scales and gives the weights for the horizontal and vertical functions, was:

$$S_{ij}=\frac{w_{i}}{X_{i}}\frac{1}{K_{j}^{2}+K_{0i}^{2}}.$$

*X_i_* sets the horizontal scale of the *i*th vertical function, and K_0i_ *=* *2/X_i_*. The values for the *X_i_* were *X_1_* = 200 km, *X_2_* = 100 km, *X_3_* = 50 km, *X_4_* = 30 km, and *X_5_* = 10 km. The horizontal length scale is shorter for higher-order vertical functions. The w_i_ were set by the spectrum of the vertical functions [13,6].

This model (vertical functions, truncated Fourier series in the horizontal, associated model variances) was used to fit the acoustic travel time data using the stochastic inverse. The prior weights of the model parameters, that is, the assumed the variances of the model parameters, *A_ij_*, are an essential component of the ocean model. These weights were defined by the wavenumber spectra.

### 2.5 Inverse procedure

The steps to computing an inverse of the Fram Strait tomography data and subsequent temperature estimate are:

1. Compute climatological sound speed and obtain a climatological sound speed section along the acoustic path at ca. 20 km intervals. This was sufficient as the World Ocean Atlas climatology is smooth. Smaller scales are described the stochastic mesoscale realization in step 2.
2. Compute a stochastic mesoscale realization for the acoustic path, as prescribed above and form the reference ocean as the sum of climatological and mesoscale environments. The reference sound speed section is:
  $$c\left(r,z\right)=c_{0}\left(r,z\right)+F_{S}\left(r,z\right).$$
3. Compute acoustic ray travel times and paths for this section between the source and receiver. Separate ray computations are required for each receiver; on some moorings there were receivers at several depths. Use rays that do not interact with the sea floor. Rays were computed using initial ray angles between −8 and 8°. Some 500–600 rays are usually obtained to each receiver as a consequence of the mesoscale variability and background sound speed profile.
4. The forward problem matrix, or operator, G, is computed as the integral over the ray paths of the 2-D model functions:
  $$G_{ij}=-2\int_{\Gamma_{i}}\frac{P_{n}\left(r,z\right)}{c_{0}^{2}\left(r,z\right)}ds.$$

Where the *Γ_j_* are the acoustic ray paths and *c_0_(r, z)* is the reference ocean sound speed section. This integral is computed for all ca. 500 rays obtained.

5. Select 5–10 of the largest peaks from within the main pulse of an arrival pattern of a transmission to use as data.
6. Randomly select 5–10 of the predicted ray travel times and their associated forward problem matrix from the “pool” of ca. 500 available eigenrays.
7. Randomly match the predicted travel times to the measured peak travel times, together with the associated forward problem matrix elements.
8. Compute inverse to solve for the amplitudes, *Â_n_* (the hat denotes estimated values) of the *P_n_(r, z)*, using 100 ms as the data uncertainty. The data used for the inverse are the measured travel times less the predicted travel times. In matrix notation where matrices are bold font.
  d=GA+ε

Where *ε* is the data uncertainty (actually a covariance but assumed diagonal).

9. Compute the corrected sound speed section:
  $$c\left(r,z\right)=c_{0}\left(r,z\right)+F_{S}\left(r,z\right)+\delta c\left(r,z\right).$$

Where:

$$\delta c\left(r,z\right)=\sum_{n=1}^{N}{\hat{A}}_{n}P_{n}\left(r,z\right).$$

10. With this solution for the sound speed section, verify that it gives travel times that are consistent with the observations.

### 2.6 Computing range- and depth-average sound speed and temperature

For computational efficiency, the average quantities were computed not in physical space, but in model space. The reason is that the averages of the *P_n_(r, z)* in:

$$F\left(r,z\right)=\sum_{n=1}^{N}{\hat{A}}_{n}P_{n}\left(r,z\right)=\sum_{i=1}^{N_{V}}\sum_{j=1}^{N_{H}}{\hat{A}}_{ij}V_{i}\left(z\right)H_{j}\left(r\right)$$

Can be computed directly, and those averages can be directly applied to the **Â**. The depth averages of the vertical functions were computed numerically, while the horizontal averages of the sines and cosines were computed analytically. As noted in [8], the ray sampling in Fram Strait is mostly in the upper 1000 m of ocean, hence averages were computed over this depth range.

The estimates for sound speed can be converted to temperature estimates using a scale factor of 4.5 m s^−1^°C^−1^. Salinity variations do not significantly affect the estimates of sound speed in Fram Strait [14].

### 2.7 List of variables

*A_ij_^1^,A_ij_^2^*: amplitudes of horizontal sinusoids in reference ocean model and inverse model
*A_n_*: amplitudes of 2-D functions in reference ocean model and inverse model
*Â_n_*: estimated amplitudes of 2-D functions by inversion
*C(z_1_,z_2_)*: vertical covariance matrix of ocean variability in reference ocean model and inverse model
c_0_(r,z): climatological sound speed section
c(r,z): sound speed section in reference ocean, updated sound speed section after inversion
d: data used for inverse
*F_s_(r, z)*: ocean state of a scalar variable (here: sound speed) in reference ocean model
*F_sI_(r, z)*: ocean state of a scalar variable (here: sound speed) in inverse model
G_ij_: forward problem matrix for inversion
H_j_(r): horizontal function in reference ocean model and inverse model, typically sinusoidal
K_0i_: horizontal scale associated with the *i*th vertical functions
K_j_: wave number of horizontal function in reference ocean model and inverse model
L: horizontal length of acoustic section
N_H_: number of horizontal functions in inverse model
N_V_: number of vertical functions in inverse model
P_n_(r,z): 2-dimensional function in reference ocean model and inverse model
*p_n_*: random number with Gaussian distribution and unit standard deviation
r: horizontal distance
*S_ij_*: wavenumber spectrum of horizontal variability
*V_i_(z)*: vertical functions in reference ocean model and inverse model
*w_i_*: overall level of wavenumber spectrum associated with each vertical function
W_n_: variance associated with the *n*th 2-D functions in reference ocean model and inverse model
X_i_: horizontal scale of i th vertical function
z: depth
*Γ_j_*: set of acoustic ray paths
δ_c_(r,z): sound speed correction from inverse
*ε*: data uncertainty in inversion

## CRediT authorship contribution statement

**F. Geyer:** Investigation, Validation, Writing – original draft, Writing – review & editing, Visualization. **H. Sagen:** Conceptualization, Resources, Writing – original draft, Writing – review & editing, Supervision, Project administration, Funding acquisition. **B. Dushaw:** Methodology, Software, Formal analysis, Investigation, Validation, Writing – original draft, Writing – review & editing, Project administration, Funding acquisition. **A. Yamakawa:** Software, Data curation, Writing – review & editing, Visualization. **M. Dzieciuch:** Resources, Writing – review & editing, Project administration. **T. Hamre:** Data curation, Writing – review & editing, Supervision, Project administration, Funding acquisition.

## Declaration of Competing Interest

The authors declare that they have no known competing financial interests or personal relationships which have, or could be perceived to have, influenced the work reported in this article.

## Data Availability

Ram Strait/ACOBAR: Range and depth average sea water temperature from acoustic tomography measurements - Fram Strait - Sep 2010 - Jul 2011 and Sep 2011 - Jul 2012 (Original data) (Norwegian Marine Data Center).
